# GLITTER: a web-based application for gene link inspection through tissue-specific coexpression

**DOI:** 10.1038/srep33460

**Published:** 2016-09-14

**Authors:** Xiangtao Liu, Pengfei Yu, Chao Cheng, James B. Potash, Shizhong Han

**Affiliations:** 1 Department of Psychiatry, University of Iowa, Iowa City, Iowa, USA; 2 Department of Bioinformatics, Admera Health LLC, South Plainfield, NJ, USA; 3 Department of Genetic, Dartmouth College, Hanover, NH, USA; 4 Interdisciplinary Graduate Program in Genetics, University of Iowa, Iowa City, Iowa, USA

## Abstract

Accumulating evidence supports the polygenic nature of most complex diseases, suggesting the involvement of many susceptibility genes with small effect sizes. Although hundreds of genes may underlie the genetic architecture of complex diseases, those involved in a given disease are probably not randomly distributed, but likely to be functionally related. Protein-protein interaction networks have been used to evaluate the functional relatedness of susceptibility genes. However, these networks do not account for tissue specificity, are limited to protein-coding genes, and are typically biased by incomplete biological knowledge. Here, we present Gene Link Inspector Through Tissue-specific coExpRession (GLITTER), a web-based application for assessing the functional relatedness of susceptibility genes, either coding or noncoding, according to tissue-specific gene expression profiles. GLITTER can also shed light on the specific tissues in which susceptibility genes might exert their functions. We further demonstrate examples of how GLITTER can evaluate the functional relatedness of susceptibility genes underlying schizophrenia and breast cancer, and provide clues about etiology.

Accumulating evidence supports the polygenic nature of most complex diseases, suggesting the involvement of many susceptibility genes with effect sizes that are typically very small^1^,^2^,^3^. Although hundreds of genes may underlie the genetic architecture of complex diseases, those involved in a given disease are probably not randomly distributed, but tend to be functionally related^4^,^5^. To evaluate this relatedness of disease susceptibility genes, protein-protein interaction (PPI) networks have been used^6^. However, PPI networks do not account for tissue specificity, are limited to protein-coding genes, and are typically biased by incomplete biological knowledge.

Complex diseases caused by susceptibility genes often show tissue-specific pathology. We reason that tissue-specific gene relationships may more closely reflect the functional relatedness of susceptibility genes than does a general PPI network. Here, we present the Gene Link Inspector through Tissue-specific coExpRession (GLITTER), a web-based application for assessing the functional relatedness of disease susceptibility genes, either coding or noncoding, according to tissue-specific gene expression profiles. GLITTER quantifies the functional relatedness of susceptibility genes by calculating the total number of connected gene pairs based on gene coexpression patterns. Its statistical significance is estimated by randomly sampled gene sets, while matching for gene numbers, gene sizes and GC content. GLITTER may also shed light on specific tissues in which susceptibility genes might exert their functions. A web-based application to implement GLITTER is freely available at http://han-lab.org/GLITTER.

## Methods

### An overview of GLITTER

**Figure 1:**
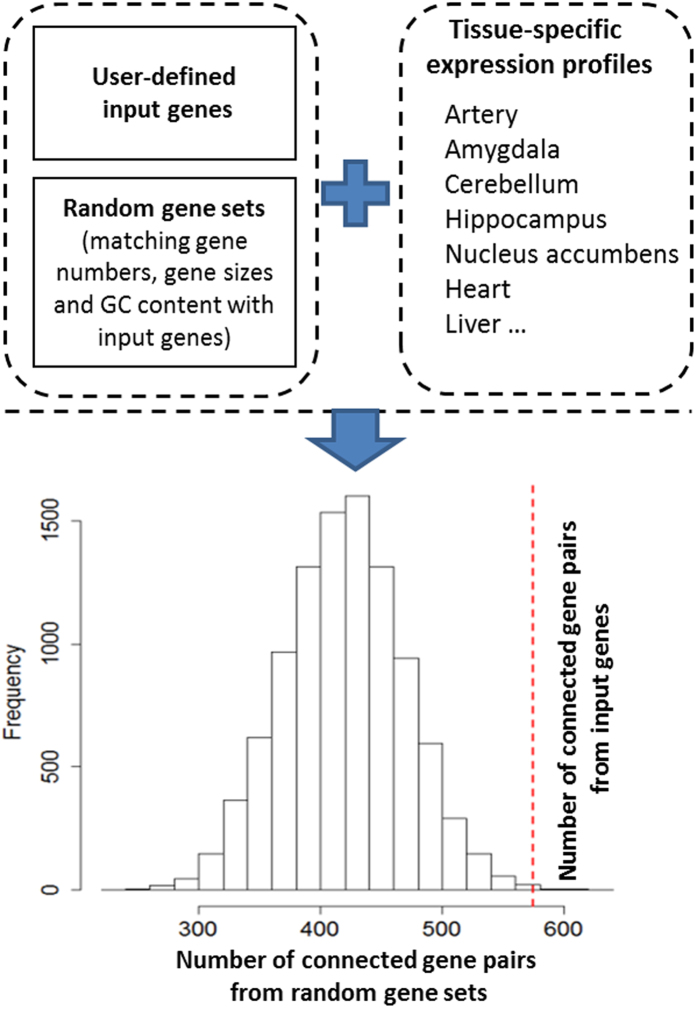
Overview of GLITTER. Given an input gene list and a set of random genes, GLITTER calculates the number of connected gene pairs of input genes. It estimates the probability of observing the same or a greater number of connected gene pairs from random gene sets than that from input genes.

An overview of GLITTER is provided in Fig. 1. The basic premise is that if two genes are highly co-expressed, then there is a good chance that they are functionally related. Based on the gene expression data sets from the Genotype-Tissue Expression (GTEx) project^7^, GLITTER evaluates the functional relatedness of user-provided input genes, either coding or non-coding, in 49 distinct tissues. Specifically, GLITTER calculates the total number of connected pairs of input genes for each tissue. Gene pairs are defined as functionally related or connected if the absolute value of their gene expression correlation is larger than a predefined threshold. To evaluate the extent to which the observed functional relatedness may occur by chance, GLITTER generates a background distribution for the number of connected gene pairs from random gene sets, matching for gene numbers, gene sizes and GC content with those of input genes. The statistical significance is then the proportion of random gene sets that show the same or a greater number of connected gene pairs than that of input genes.

### The GTEx data resource

We downloaded RNA-Seq data (version 6), summarized to Gencode v19 gene-level reads per kilobase million mapped reads (RPKM) values, from the GTEx Portal website. Detailed information on tissue processing, experimental and bioinformatics procedures related to the RNA-Seq data is available at http://www.gtexportal.org/home/. We limited our analysis to 49 distinct tissues that each had sample size >20, including 13 brain region-specific tissues and 36 non-brain tissues. For each tissue, we considered genes expressed if they had a normalized RPKM value of ≥1 for at least 80% of the samples. Gene expression values were log-transformed (log_2_ [RPKM + 1]) for gene co-expression analysis.

### Functional relatedness of susceptibility genes

Utilizing the gene expression data from GTEx, we used gene coexpression patterns to evaluate the functional relatedness of susceptibility genes. GLITTER provides two options, Pearson correlation or biweight midcorrelation, for calculating gene expression correlations between two genes. Biweight midcorrelation is more robust against outliers than Pearson correlation^8^, and is computed using the *bicor* function in the R-package *WGCNA*^9^. Given a predefined correlation threshold, GLITTER calculates the total number of connected gene pairs among susceptibility genes. The statistical significance is estimated by generating a background distribution for the number of connected gene pairs within random gene sets, while matching for gene numbers, gene sizes and GC content based on the features found in the susceptibility gene list. The matching of random gene sets was achieved by first partitioning reference genes into bins of different gene length and GC content, followed by counting the number of input genes within each bin. Random gene sets were then generated by randomly sampling each bin, and choosing the same number of genes from each as that of input genes that fell into each bin. The p-value for the functional relatedness of input genes is then the proportion of random gene sets that achieves the same or a greater number of connected gene pairs in each tissue, as compared to the number found within the susceptibility gene set.

### Input and output

GLITTER takes a list of user-defined genes as input, together with options for the type of correlation, correlation threshold, and the number of simulations for random gene sets. The gene names can either be official symbols or ENSEMBLE IDs. The two correlation-related options (correlation type and correlation threshold) determine the way to estimate the connections of genes in each tissue. Simulations are used for estimating the significance of the observed number of connected gene pairs among input genes. Random gene sets are generated from the human Gencode v19 gene annotation by default. The users can also choose to generate random gene sets from customized gene sets, such as those represented in a GWAS array. We note that the customized reference gene sets should contain as many genes as possible, so that the background distribution can be approximated by random gene sets. GLITTER returns two sets of results: (1) a summary text file with each line containing meta information for each tissue, including tissue name, total number of expressed genes, the number of input genes expressed, the number of connected gene pairs among input genes, and the p-value assessing the significance of the observed number of connected gene pairs; and (2) a series of PDF files for the histogram of the number of connected gene pairs from random gene sets for each tissue. The running time depends on the number of input genes and other selected parameters. For a set of 102 genes and 10,000 simulations, it takes about one hour. GLITTER sends an email notice to users once the job is done, alerting them that the results are ready to be downloaded from the website.

## Results

**Figure 2:**
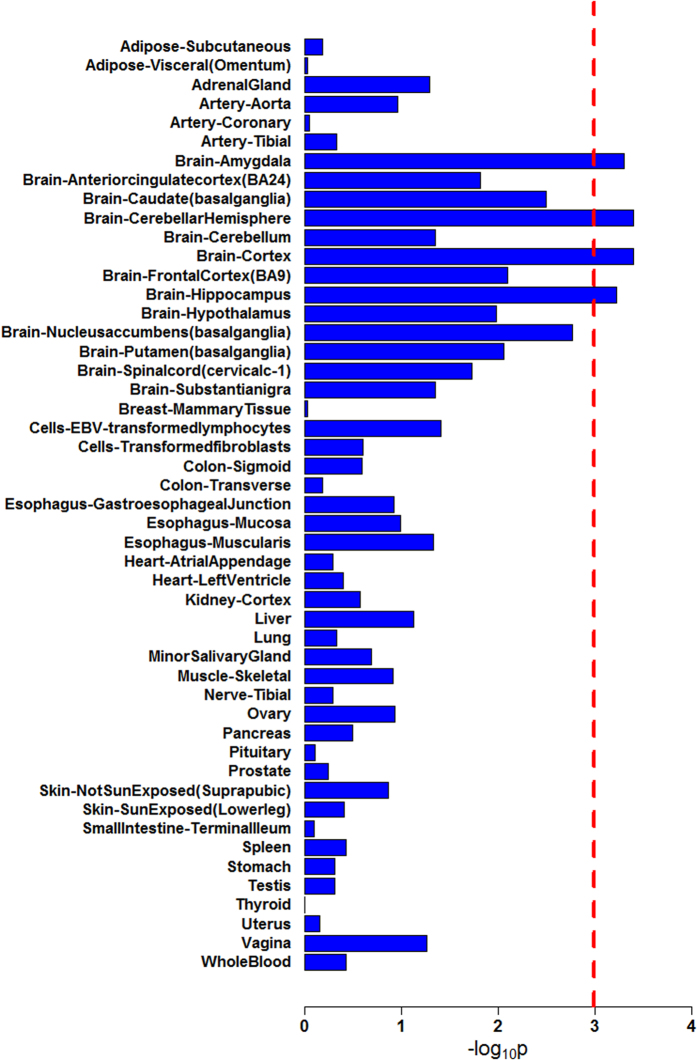
Functional relatedness analysis for schizophrenia susceptibility genes in 49 tissues. The x-axis is the –log (p-value) that measures the likelihood that we would observe the same or a greater number of connected gene pairs from random gene sets, compared to the number of connected gene pairs among schizophrenia genes. The y-axis labels the different tissues examined. The dotted red line indicates the significance threshold after Bonferroni correction for the number of tissues examined.

We applied GLITTER to evaluate the functional relatedness of 97 schizophrenia candidate genes implicated by the 108 independent schizophrenia risk loci identified in a recent genome-wide association study of schizophrenia from the Psychiatric Genomics Consortium (PGC)^10^ (see full list of candidate genes in Supplementary Table S1). If risk loci contained multiple genes, we prioritized genes using the Summary-data-based Mendelian Randomization (SMR) method^11^. SMR is a useful tool developed to prioritize genes underlying GWAS hits by integrating data from both GWAS and expression quantitative trait loci (eQTL) studies. Specifically, we downloaded from the SMR website the gene-based p-values of the PGC schizophrenia GWAS data derived from the SMR method. For risk loci with multiple genes, we then selected the gene with the smallest SMR method-based p-value as the candidate risk gene. Gene pairs were connected if the absolute value of the biweight midcorrelation of their gene expression was ≥0.7. We used 10,000 simulations to estimate the significance of the observed number of connected gene pairs. Random gene sets were selected from the 51,746 genes covered by the imputed data of PGC. Figure 2 shows the p-values of the functional connectedness among schizophrenia genes in 49 tissues. Detailed summary statistics for each tissue are presented in Supplementary Table S2. We found that schizophrenia genes tend to be more connected than random gene sets in a number of brain regions, and this result remained significant after Bonferroni correction (p_corrected_ ≤ 0.05), suggesting their potentially important roles in the etiopathogenesis of the disease. These regions included the amygdala (p = 0.0005), cerebellar hemisphere (p = 0.0004), cortex (p = 0.0004), and hippocampus (p = 0.0006). However, we did not observe a similar pattern in non-brain tissues. These data strongly support the functional relatedness of schizophrenia genes in brain-related tissues, but not in other tissues.

To evaluate the generalizability of GLITTER, we also applied it to examine the functional relatedness of 49 breast cancer susceptibility genes identified through GWAS^12^ (see full list of candidate genes in Supplementary Table S3). Similarly, we defined gene pairs as connected if the absolute value of the biweight midcorrelation of their gene expression was ≥0.7. We used 10,000 simulations to estimate the significance of connected gene pairs. Interestingly, we found that among all tissues examined, the breast mammary tissue showed the strongest statistical evidence for non-random connectedness among breast cancer genes (p = 0.0015), and this result was nearly significant after Bonferroni correction (p_corrected_ = 0.07). There were only two other tissues with nominal levels of evidence for the non-random connectedness of breast cancer genes: kidney cortex (p = 0.03) and uterus (p = 0.04). Supplementary Table S4 provides the detailed summary statistics from GLITTER for each tissue.

Finally, as a comparison between GLITTER and an existing method of assessing the functional relatedness of gene sets, we used the Disease Association Protein-protein Link Evaluator (DAPPLE)^6^. We examined whether the protein products of the above susceptibility genes interact with each other more often than expected. We observed seven and zero direct connections for schizophrenia (p = 0.59) and breast cancer genes (p = 1), respectively, which were not statistically significant. To further quantify associations among disease genes, we extended the network metrics from direct connections to several other metrics, including associated protein direct connectivity, associated protein indirect connectivity, and common interactor connectivity as reported by DAPPLE. However, we still did not observe any significant results for those network metrics among either schizophrenia or breast cancer genes (p > 0.05).

## Discussion

In summary, we designed GLITTER, a user-friendly web-based tool, to examine the functional relatedness of susceptibility genes according to tissue-specific gene expression profiles. GLITTER employs the basic assumption that if two genes are highly co-expressed then there is a good chance they are functionally related. GLITTER first estimates the functional relatedness of susceptibility genes by counting the total number of connected gene pairs based on gene coexpression patterns. It then uses simulations to estimate the likelihood that the same or a greater number of connected gene pairs can be observed from random gene sets, while matching for basic features (gene numbers, gene sizes and GC content) found in the susceptibility gene list.

The motivation underlying GLITTER is based on the fact that complex diseases often show tissue-specific pathology. We reason that gene relationships in the context of disease-relevant tissues may more closely reflect the functional relationships among susceptibility genes than those captured in a tissue-independent context through the general PPI network. Compared to PPI networks, gene co-expression networks are less biased by incomplete biological knowledge. Therefore, GLITTER has the advantage of comprehensiveness in defining all potential functionally related gene pairs. In addition, GLITTER can also shed light on specific tissues in which susceptibility genes might exert their functions and therefore enhance our understanding of disease etiology. We note that GLITTER is especially useful for evaluating susceptibility genes underlying psychiatric disorders, because it allows evaluation of gene connections in a number of brain regions, and thus provides opportunities to look into specific brain regions that may be related to underlying disease pathophysiology. Our case study illustrates how GLITTER can evaluate the functional relatedness of schizophrenia susceptibility genes and provide clues about etiopathogenesis. The case study of breast cancer genes further demonstrates the generalizability of GLITTER to other complex diseases.

We note that GLITTER has also some limitations. For example, GLITTER uses a random sampling approach for estimating the statistical significance of connected gene pairs. Although random gene sets match for some basic features found in the input genes, such as gene numbers, gene sizes and GC content, the random sampling approach cannot fully account for all important features that determine the background distribution of the test statistic. In addition, while the gene co-expression network approach has the advantage of comprehensiveness, as it captures all potential functionally related gene pairs, it may also inevitably introduce a greater amount of noise compared to the PPI network approach. Therefore, we view GLITTER as by no means a replacement for current tools, such as DAPPPLE, but as a complementary tool for assessing the functional relationships among sets of potential disease genes.

## Additional Information

**How to cite this article**: Liu, X. *et al*. GLITTER: a web-based application for gene link inspection through tissue-specific coexpression. *Sci. Rep.* **6**, 33460; doi: 10.1038/srep33460 (2016).

## Supplementary Material

Supplementary Information
