# Supplementary material for: GLITTER: a web-based application for gene link inspection through tissue-specific coexpression
Source: Sci Rep. 2016 Sep 14;6:33460. doi: 10.1038/srep33460 (PMC5022062; doi:10.1038/srep33460)
Supplement: Supplementary Information [file srep33460-s1.pdf]

GLITTER: a web-based application for gene link inspection through tissue-specific coexpression

Xiangtao Liu[1, †], Pengfei Yu[2, †], Chao Cheng[3], James B. Potash[1, 4] and Shizhong Han[1, 4*]

1) Department of Psychiatry, University of Iowa, Iowa City, Iowa, USA
2) Department of Bioinformatics, Admera Health LLC, South Plainfield, NJ, USA
3) Department of Genetic, Dartmouth College, Hanover, NH, USA
4) Interdisciplinary Graduate Program in Genetics, University of Iowa, Iowa City, Iowa, USA

Supplementary Table S1. Basic information for 97 schizophrenia candidate genes from the Psychiatric Genomics Consortium genome-wide association study of schizophrenia

CHR	START	END	ENSEMBL ID	STRAND	GENE SYMBOL
chr7	1855429	2272878	ENSG00000002822.11	-	MAD1L1
chr2	72356367	72375167	ENSG00000003137.4	-	CYP26B1
chr11	123396344	123498482	ENSG00000023171.10	+	GRAMD1B
chr1	150121373	150136916	ENSG00000023902.9	+	PLEKHO1
chr2	58134786	58387055	ENSG00000028116.12	+	VRK2
chr2	225334867	225450110	ENSG00000036257.8	-	CUL3
chr15	78832747	78841604	ENSG00000041357.11	+	PSMA4
chr1	243419320	243663394	ENSG00000054282.11	+	SDCCAG8
chr6	84262599	84419410	ENSG00000065609.10	-	SNAP91
chr17	17713713	17740325	ENSG00000072310.12	-	SREBF1
chr10	104845940	104953056	ENSG00000076685.14	-	NT5C2
chr6	72596406	73112845	ENSG00000079841.14	+	RIMS1
chr11	133778459	133826880	ENSG00000080854.10	-	IGSF9B
chr5	88013975	88199922	ENSG00000081189.9	-	MEF2C
chr7	105080108	105162714	ENSG00000091127.9	-	PUS7
chr22	39966758	40085742	ENSG00000100346.13	+	CACNA1I
chr22	41601209	41627275	ENSG00000100395.10	+	L3MBTL2
chr20	37434348	37551667	ENSG00000101445.5	+	PPP1R16B
chr7	136912088	137028611	ENSG00000105894.7	-	PTN
chr7	24737972	24809244	ENSG00000105928.9	-	DFNA5
chr4	170533784	170644824	ENSG00000109572.9	+	CLCN3
chr5	109025067	109205326	ENSG00000112893.5	+	MAN2A1
chr3	135969148	136056738	ENSG00000114054.9	+	PCCB
chr3	180585929	180700541	ENSG00000114416.13	+	FXR1
chr3	52738971	52742182	ENSG00000114902.9	+	SPCS1
chr2	198254508	198299815	ENSG00000115524.11	-	SF3B1
chr2	200134223	200335989	ENSG00000119042.12	-	SATB2
chr5	140044261	140053709	ENSG00000120314.14	+	WDR55
chr11	130745331	130786404	ENSG00000120451.6	-	SNX19
chr5	137841784	137878989	ENSG00000120705.8	-	ETF1
chr8	27348296	27403081	ENSG00000120915.9	+	EPHX2
chr16	58553855	58663790	ENSG00000125107.12	-	CNOT1
chr14	99635624	99737861	ENSG00000127152.13	-	BCL11B
chr7	131185021	131242976	ENSG00000128567.12	-	PODXL
chr5	60628100	60841997	ENSG00000130449.5	+	ZSWIM6
chr3	17198654	18486309	ENSG00000131374.10	-	TBC1D5
chr12	29653773	29937692	ENSG00000133687.11	-	TMTC1
chr2	149402009	149545130	ENSG00000135999.7	+	EPC2

chr15	40509629	40569688	ENSG00000137843.7	+	PAK6
chr4	103172198	103352415	ENSG00000138821.8	-	SLC39A8
chr15	70340129	70390515	ENSG00000140332.11	-	TLE3
chr15	91411822	91426688	ENSG00000140564.6	+	FURIN
chr19	50030875	50050219	ENSG00000142552.3	+	RCN3
chr1	8412457	8877702	ENSG00000142599.13	-	RERE
chr1	43990858	44089343	ENSG00000142949.12	+	PTPRF
chr2	162280843	162841792	ENSG00000144290.12	+	SLC4A10
chr3	2140497	3099645	ENSG00000144619.10	+	CNTN4
chrX	5758678	6146904	ENSG00000146938.10	-	NLGN4X
chr11	113280318	113346413	ENSG00000149295.9	-	DRD2
chr1	2357419	2436969	ENSG00000149527.13	+	PLCH2
chr11	124622026	124632186	ENSG00000149564.7	-	ESAM
chrX	21392536	21672813	ENSG00000149970.10	+	CNKSR2
chr4	176554085	176923815	ENSG00000150625.12	-	GPM6A
chr12	123405498	123466196	ENSG00000150967.13	-	ABCB9
chr12	2079952	2802108	ENSG00000151067.16	+	CACNA1C
chr5	152869175	153193429	ENSG00000155511.13	+	GRIA1
chr8	89044237	89340254	ENSG00000156103.11	-	MMP16
chr11	57435219	57468659	ENSG00000156599.6	+	ZDHHC5
chr20	47980414	48099184	ENSG00000158445.7	-	KCNB1
chr1	74491699	74663871	ENSG00000162620.11	-	LRRIQ3
chr3	63850233	63989138	ENSG00000163635.13	+	ATXN7
chr5	153570290	153800544	ENSG00000164574.11	+	GALNT10
chr5	45259349	45696253	ENSG00000164588.4	-	HCN1
chr8	110975874	110988076	ENSG00000164794.4	-	KCNV1
chr10	18429606	18830798	ENSG00000165995.14	+	CACNB2
chr14	103985996	103989448	ENSG00000166165.8	-	CKB
chr12	57489191	57525922	ENSG00000166888.6	-	STAT6
chr19	19496635	19619740	ENSG00000167491.13	+	GATAD2A
chr17	2206677	2228554	ENSG00000167720.8	+	SRR
chr3	36868311	36986548	ENSG00000168016.9	-	TRANK1
chr2	185463093	185804219	ENSG00000170396.6	+	ZNF804A
chr8	143293441	143484601	ENSG00000171045.10	-	TSNARE1
chr6	96463860	96663488	ENSG00000172461.6	+	FUT9
chr12	110718561	110788898	ENSG00000174437.12	+	ATP2A2
chr16	29916333	29938356	ENSG00000174943.5	-	KCTD13
chr16	14014014	14046202	ENSG00000175595.10	+	ERCC4
chr8	61099906	61193971	ENSG00000178538.5	-	CA8
chr12	103631369	103889749	ENSG00000179088.10	-	C12orf42
chr11	46406640	46408107	ENSG00000180720.6	-	CHRM4

chrX	68380694	68385636	ENSG00000181191.11	-	PJA1
chr14	72399156	73030654	ENSG00000182732.12	+	RGS6
chr8	2792875	4852494	ENSG00000183117.13	-	CSMD1
chr16	9852376	10276611	ENSG00000183454.9	-	GRIN2A
chr14	30045687	30661104	ENSG00000184304.10	-	PRKD1
chr7	110303110	111202573	ENSG00000184903.5	-	IMMP2L
chr11	109292846	109299840	ENSG00000185742.6	+	C11orf87
chr11	24518516	25104150	ENSG00000187398.7	+	LUZP2
chr1	97543299	98386605	ENSG00000188641.8	-	DPYD
chr18	52889562	53332018	ENSG00000196628.9	-	TCF4
chr9	84198598	84304220	ENSG00000196781.9	-	TLE1
chr19	30719197	31204445	ENSG00000198597.4	+	ZNF536
chr7	86273230	86494200	ENSG00000198822.6	+	GRM3
chr22	42454358	42466846	ENSG00000198951.7	-	NAGA
chr2	233562009	233725285	ENSG00000204120.10	+	GIGYF2
chrX	37883148	37883239	ENSG00000221466.2	-	MIR548AJ2
chr2	193614571	193641625	ENSG00000227418.2	+	PCGEM1
chr12	92378756	92536690	ENSG00000257242.2	-	C12orf79

Supplementary Table S2. Detailed summary statistics of the functional relatedness analysis of schizophrenia candidate genes by GLITTER

Tissue	Number of total expressed genes	Number of expressed input genes	Number of connections	P-value	Bonferroni adjusted p-value
Adipose-Subcutaneous	23422	90	2	0.66	1.00
Adipose-Visceral(Omentum)	23409	90	11	0.95	1.00
AdrenalGland	23319	90	8	0.050	0.92
Artery-Aorta	22957	86	5	0.10	1.00
Artery-Coronary	23266	89	3	0.89	1.00
Artery-Tibial	22091	88	4	0.47	1.00
Brain-Amygdala	23602	95	504	5.00E-04	0.02
Brain-Anteriorcingulatecortex(BA24)	24143	95	779	0.015	0.53
Brain-Caudate(basalganglia)	24400	94	637	0.0032	0.15
Brain-CerebellarHemisphere	24518	93	120	4.00E-04	0.02
Brain-Cerebellum	24867	93	31	0.044	0.89
Brain-Cortex	24769	95	303	4.00E-04	0.02
Brain-FrontalCortex(BA9)	24370	95	488	0.0081	0.33
Brain-Hippocampus	23508	95	486	6.00E-04	0.03
Brain-Hypothalamus	24453	95	513	0.010	0.40
Brain-Nucleusaccumbens(basalganglia)	24385	94	653	0.0017	0.08
Brain-Putamen(basalganglia)	23707	94	845	0.0088	0.35
Brain-Spinalcord(cervicalc-1)	23562	93	226	0.018	0.61
Brain-Substantianigra	23473	93	267	0.044	0.89
Breast-MammaryTissue	23980	92	32	0.95	1.00
Cells-EBV-transformedlymphocytes	22393	80	7	0.039	0.86
Cells-Transformedfibroblasts	21392	77	57	0.25	1.00
Colon-Sigmoid	23512	91	14	0.26	1.00
Colon-Transverse	23917	91	373	0.66	1.00
Esophagus-GastroesophagealJunction	23278	90	14	0.12	1.00
Esophagus-Mucosa	23331	87	29	0.10	0.99
Esophagus-Muscularis	23288	89	15	0.047	0.91
Heart-AtrialAppendage	22708	89	10	0.52	1.00
Heart-LeftVentricle	21487	84	57	0.40	1.00
Kidney-Cortex	24154	88	249	0.27	1.00
Liver	21837	78	14	0.075	0.98
Lung	24386	91	7	0.47	1.00

MinorSalivaryGland	24812	90	114	0.20	1.00
Muscle-Skeletal	20722	86	18	0.12	1.00
Nerve-Tibial	24669	92	4	0.51	1.00
Ovary	24046	87	6	0.11	1.00
Pancreas	22902	89	78	0.32	1.00
Pituitary	25845	95	2	0.78	1.00
Prostate	25126	92	7	0.58	1.00
Skin-NotSunExposed(Suprapubic)	23991	90	18	0.13	1.00
Skin-SunExposed(Lowerleg)	23908	91	9	0.39	1.00
SmallIntestine-TerminalIleum	25012	92	170	0.80	1.00
Spleen	24687	87	6	0.37	1.00
Stomach	22978	90	204	0.48	1.00
Testis	33948	95	71	0.48	1.00
Thyroid	24671	89	0	1	1.00
Uterus	24089	86	8	0.70	1.00
Vagina	24164	92	281	0.057	0.94
WholeBlood	19202	77	189	0.37	1.00

Supplementary Table S3. Basic information for 49 breast cancer genes identified through GWAS

CHR	START	END	ENSEMBL ID	STRAND	GENE SYMBOL
chr14	91737667	91884188	ENSG00000015133.14	-	CCDC88C
chr3	27414214	27525911	ENSG00000033867.12	-	SLC4A7
chr7	144052381	144077725	ENSG00000050327.10	+	ARHGEF5
chr6	149539777	149732749	ENSG00000055208.13	+	TAB2
chr1	203764782	203823252	ENSG00000058673.11	+	ZC3H11A
chr10	123237848	123357972	ENSG00000066468.16	-	FGFR2
chr6	151977826	152450754	ENSG00000091831.17	+	ESR1
chr19	17378159	17392058	ENSG00000105393.11	+	BABAM1
chr19	18553473	18632937	ENSG00000105656.8	-	ELL
chr10	80828792	81076276	ENSG00000108175.12	+	ZMIZ1
chr5	58264865	59817947	ENSG00000113448.12	-	PDE4D
chr1	114447763	114456708	ENSG00000118655.4	+	DCLRE1B
chr6	151815165	151942328	ENSG00000120262.8	+	CCDC170
chr20	32581452	32696114	ENSG00000125970.7	+	RALY
chr7	91570181	91739987	ENSG00000127914.12	+	AKAP9
chr11	1874200	1913497	ENSG00000130592.9	+	LSP1
chr11	1940792	1959936	ENSG00000130595.12	+	TNNT3
chr1	202163029	202288909	ENSG00000133067.13	+	LGR6
chr10	64133951	64431771	ENSG00000138311.11	+	ZNF365
chr13	32889611	32973805	ENSG00000139618.10	+	BRCA2
chr16	53737875	54155853	ENSG00000140718.14	+	FTO
chr1	10532345	10690815	ENSG00000142655.8	+	PEX14
chr9	22002902	22009362	ENSG00000147883.9	-	CDKN2B
chr10	114710009	114927437	ENSG00000148737.11	+	TCF7L2
chr3	4535032	4889524	ENSG00000150995.13	+	ITPR1
chr18	24495595	24765281	ENSG00000154080.8	-	CHST9
chr19	17392454	17398455	ENSG00000160117.10	+	ANKLE1
chr3	30647994	30735634	ENSG00000163513.13	+	TGFBR2
chr3	63850233	63989138	ENSG00000163635.13	+	ATXN7
chr5	158122928	158526769	ENSG00000164330.12	-	EBF1
chr5	1253262	1295184	ENSG00000164362.14	-	TERT
chr8	76320149	76479078	ENSG00000164749.7	+	HNF4G
chr17	53046088	53241646	ENSG00000166263.9	+	STXBP4
chr16	80631803	80838226	ENSG00000166446.10	-	CDYL2
chr4	175750819	175899331	ENSG00000168594.11	+	ADAM29
chr4	106067032	106200973	ENSG00000168769.8	+	TET2
chr2	212240446	213403565	ENSG00000178568.9	-	ERBB4
chr14	68286496	69196935	ENSG00000182185.14	+	RAD51B
chr22	29601840	29655586	ENSG00000186998.11	+	EMID1

chr22	40806285	41032706	ENSG00000196588.10	-	MKL1
chr1	204485511	204542871	ENSG00000198625.8	+	MDM4
chr14	37126773	37148920	ENSG00000198807.8	+	PAX9
chr15	91509270	91538859	ENSG00000198901.9	-	PRC1
chr2	218148742	218621316	ENSG00000231672.2	-	DIRC3
chr1	121261143	121310591	ENSG00000231752.1	+	EMBP1
chr8	128302062	128494384	ENSG00000246228.2	-	CASC8
chr16	52586002	52686017	ENSG00000249231.3	-	CASC16
chr8	76135639	76236976	ENSG00000249395.2	-	CASC9
chr13	25591541	25591675	ENSG00000269099.1	+	LSP1

Supplementary Table S4. Detailed summary statistics of the functional relatedness analysis of breast cancer candidate genes by GLITTER

Tissue	Number of total expressed genes	Number of expressed input genes	Number of connections	P-value	Bonferroni adjusted p-value
Adipose-Subcutaneous	23422	40	1	0.42	1.00
Adipose-Visceral(Omentum)	23409	40	3	0.66	1.00
AdrenalGland	23319	40	0	1	1.00
Artery-Aorta	22957	40	2	0.12	1.00
Artery-Coronary	23266	39	3	0.28	1.00
Artery-Tibial	22091	41	1	0.59	1.00
Brain-Amygdala	23602	42	38	0.64	1.00
Brain-Anteriorcingulatecortex(BA24)	24143	43	57	0.93	1.00
Brain-Caudate(basalganglia)	24400	44	63	0.68	1.00
Brain-CerebellarHemisphere	24518	42	2	0.99	1.00
Brain-Cerebellum	24867	42	1	0.95	1.00
Brain-Cortex	24769	44	11	0.89	1.00
Brain-FrontalCortex(BA9)	24370	44	27	0.94	1.00
Brain-Hippocampus	23508	41	40	0.73	1.00
Brain-Hypothalamus	24453	43	33	0.93	1.00
Brain-Nucleusaccumbens(basalganglia)	24385	44	57	0.91	1.00
Brain-Putamen(basalganglia)	23707	43	96	0.59	1.00
Brain-Spinalcord(cervicalc-1)	23562	42	29	0.77	1.00
Brain-Substantianigra	23473	44	15	0.98	1.00
Breast-MammaryTissue	23980	43	55	0.0015	0.07
Cells-EBV-transformedlymphocytes	22393	40	2	0.21	1.00
Cells-Transformedfibroblasts	21392	41	17	0.26	1.00
Colon-Sigmoid	23512	42	1	0.80	1.00
Colon-Transverse	23917	43	108	0.16	1.00
Esophagus-GastroesophagealJunction	23278	41	2	0.50	1.00
Esophagus-Mucosa	23331	44	7	0.35	1.00
Esophagus-Muscularis	23288	41	1	0.75	1.00
Heart-AtrialAppendage	22708	40	4	0.19	1.00
Heart-LeftVentricle	21487	40	6	0.92	1.00
Kidney-Cortex	24154	44	106	0.032	0.80
Liver	21837	41	1	0.90	1.00

Lung	24386	44	4	0.21	1.00
MinorSalivaryGland	24812	45	30	0.40	1.00
Muscle-Skeletal	20722	39	2	0.57	1.00
Nerve-Tibial	24669	43	2	0.24	1.00
Ovary	24046	39	1	0.49	1.00
Pancreas	22902	41	24	0.27	1.00
Pituitary	25845	45	1	0.81	1.00
Prostate	25126	42	2	0.49	1.00
Skin-NotSunExposed(Suprapubic)	23991	43	5	0.16	1.00
Skin-SunExposed(Lowerleg)	23908	42	3	0.25	1.00
SmallIntestine-TerminalIleum	25012	46	70	0.38	1.00
Spleen	24687	44	2	0.40	1.00
Stomach	22978	42	37	0.70	1.00
Testis	33948	48	15	0.62	1.00
Thyroid	24671	40	1	0.18	1.00
Uterus	24089	40	8	0.047	0.88
Vagina	24164	43	59	0.10	1.00
WholeBlood	19202	40	56	0.21	1.00
